# Uncovering the knowledge gaps: A survey on human monkeypox infection among men who have sex with men in Nepal

**DOI:** 10.3126/hprospect.v22i1.53504

**Published:** 2023-11-02

**Authors:** Kiran Paudel, Kamal Gautam, Md Safaet Hossain Sujan, Jeffrey A Wickersham, Prashu Ram Chaudhari, Roman Shrestha

**Affiliations:** 1 Nepal Health Frontiers, Tokha-5, Kathmandu, Nepal; 2 University of Connecticut, Department of Allied Health Sciences, Storrs, CT 06269, USA; 3 Yale School of Medicine, Department of Internal Medicine, Section of Infectious Diseases, 135 College St.,Suite 323, New Haven, CT 06510, USA; 4 Blue Diamond Society, Kathmandu, Nepal

**Keywords:** Monkeypox, MPOX, men who have sex with men, MSM, Nepal

## Abstract

The monkeypox virus (MPOX) poses a major threat to the health of people, particularly among men who have sex with men (MSM). However, the knowledge regarding MPOX among MSM in Nepal is poorly understood. Therefore, the present study aimed to assess the knowledge of human monkeypox among Nepalese MSM. A cross-sectional face-to-face survey was conducted among purposively selected 250 participants (mean age 27.6 ± 8.9 years) in Nepal between October and December 2022. The questionnaire consisted of informed consent along with questions covering age, education, and MPOX. The findings demonstrated that the majority of MSM in Nepal had poor knowledge of MPOX. Around 87% of participants had no idea whether MPOX is contagious or not. However, the results call for immediate action to improve knowledge of MSM through health education programs and appropriate interventions to spread awareness of MPOX.

## Tweetable Abstract

There is need for improving knowledge of MSM through health education programs and appropriate interventions to spread awareness of MPOX.

## Introduction

Human monkeypox (HMPOX) is a sporadic zoonosis caused by monkeypox virus (MPOX) infection and has been described as endemic for over 60 years in several countries in Africa [1] MPOX symptoms are often like those of smallpox but tend to be milder. These symptoms include severe headache, fever, muscle soreness, enlargement of lymph nodes, back pain, and weakness, and are mostly transmitted through sexual contact [1, 2]. As the number of incidences of MPOX is escalating in non-African countries and impacting the health of people rigorously, the World Health Organization (WHO) declared a public health emergency in July 2022 for international concerns [3].

As of 28th February 2023, WHO has reported 86,173 confirmed cases and 99 deaths from 110 different countries [4]. According to the Centers for Disease Control and Prevention (CDC), all cases of monkeypox have occurred in men (99%), and a substantial proportion of these men (94%) had sexual or intimate contact with other men. According to a recent report, apart from Nigeria, all the other nations indicated that most of the reported cases were MSM, and that most of these incidences were found among people who had multiple partners [5], and commonly met at places such as bars, sex clubs, or through dating apps and sex parties [6].

Moreover, preventive measures are considered the most effective means of controlling the spread of MPOX, which can be accomplished through vaccination, abstaining from contact with infected animals or individuals, and maintaining good hygiene practices [4, 6–8]. For this, adequate knowledge plays a pivotal role in controlling and executing MPOX awareness program. Conversely, lack of knowledge and inappropriate awareness among MSM can have detrimental effects on the control of transmission, prevention effectors, early detection, and rapid management of infections [9]. A study on MPOX information on YouTube found that 11.9% contained misleading information about its epidemiology, symptoms, testing, treatment, and transmission [10]. Thus, a study has become necessary to reveal the current scenario of knowledge among MSM to take appropriate actions to control MPOX.

To the best of the author’s knowledge, no prior research in Nepal investigated the prevalence of knowledge of MPOX among MSM. Consequently, the current study aims to explore the existing research gap.

## Methods

### Study design and participants

This cross-sectional study was conducted among MSM who were at least 18 years old, understood Nepali or English and were residing in Kathmandu valley. In our study, 250 MSM were enrolled by using Response Driven Sampling (RDS) method, from October to December 2022. RDS has been found effective for recruiting hidden and hard-to-reach population [11]. We purposively selected five MSM “seeds” based on recommendations from community-based organizations led by LGBTIQA+ and gave them 5 recruitment coupons to distribute to potential participants. Each subsequent participant was also given 5 recruitment coupons to recruit additional peers. The exclusion criteria for the study were respondents who gave incomplete and multiple answers to the survey.

### Study variables

Measures related to monkeypox knowledge were obtained by asking twenty-three items of questions as previously done [12, 13]. Each knowledge question had three possible answers: (yes, no, and I do not know). Sources of monkeypox infection, routes of transmission, susceptible group, general clinical symptoms, and preventive measures were included in the knowledge-related variables.

Additionally, participants’ age (later categorized into less than 25, and above 25), education (up to SLC, plus two and above), and smartphone usage for health purposes (never or rarely, most of the time) were also asked. The survey was conducted by the trained research assistant in the private room using Qualtrics.

### Statistical analysis

Descriptive statistics (frequencies, percentages, means, and standard deviations) were performed in this study. We calculated frequencies and percentages for categorical variables and mean and standard deviations for continuous variables. The Chi-square test was used to determine the association between independent and dependent categorical variables. The statistical significance was set at p < 0.050 as the cut-off level. All analysis was carried out by using IBM Statistical Package for the Social Sciences (SPSS), Version 26.0.

### Ethics and consent

The study was carried out by following the Institutional Research Ethics and Human Participation Criteria (Helsinki Declaration). The Nepal Health Research Council approved the present study. Prior to enrollment, all study participants were provided with comprehensive information regarding the study’s purpose, methods, and objectives, as well as their rights pertaining to privacy and anonymity of their responses, and the option to withdraw from the study at any time. Written informed consent was obtained from all participants.

## Results

A total of 250 participants were included in the study, with a mean age of 27.6 ± 8.9 years old, and majority of them were gay (63.2%) in sexual orientation (Table 1).

**Figure 1:**
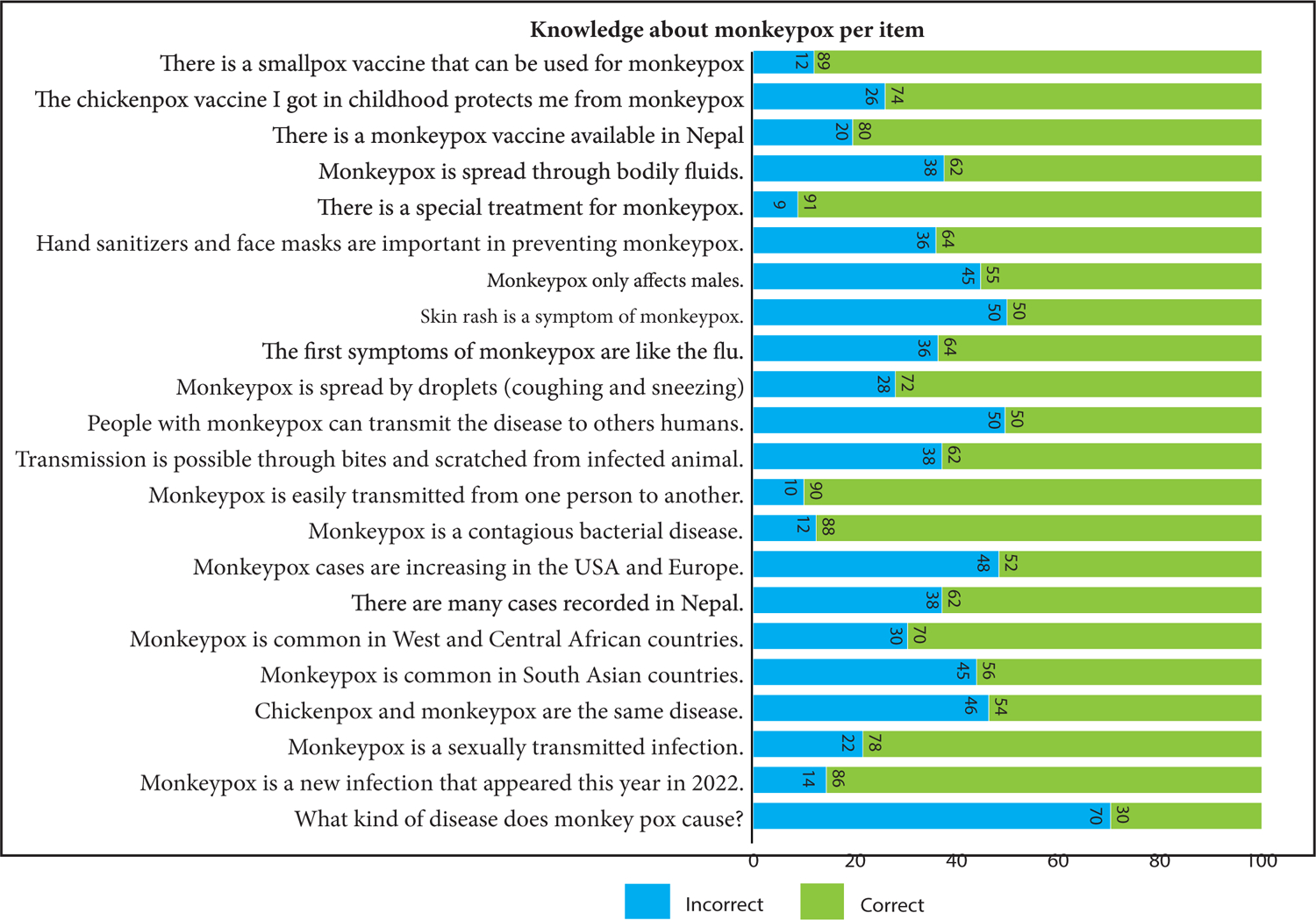
The overall level of human monkeypox knowledge among the respondents.

The overall level of knowledge regarding MPOX was poor, with only one item (What kind of disease does monkey pox cause?) having a correct response level of >70% (Figure 1). Notably, only 10% of the respondents were aware that the MPOX gets transmitted from one person to another. Additionally, 91% of participants had no idea whether MPOX had any cure.

Moreover, the differences were less conspicuous upon comparing the level of knowledge based on age. Education and use of smartphones for health-related information appeared to have a significant association with better knowledge among those who studied plus two and above and used smartphones most of the time, as shown in Table 2.

## Discussion

There is a minimal fatality rate linked to MPOX since it is a self-limiting viral illness. The WHO has deemed it a public health emergency to stimulate coordinated efforts across countries to effectively manage the disease before it spreads around the world [14].

Evaluation of MPOX related knowledge among Nepalese MSM would assist to provide the groundwork for steps to educate the MSM and the public, and include them in control, preventive, and treatment measures; hence effectively controlling and eradicating the monkeypox epidemic. This research assessed the level of monkeypox knowledge among Nepal’s MSM. The study was conducted during the monkeypox outbreak in Southeast Asia [15]. According to the present study, most MSM have poor knowledge about the disease’s transmission, symptoms and clinical differences between smallpox and chicken pox. In line with the present study, some prior studies conducted among the public of Saudi Arabia, and Indonesia revealed the similar findings [13, 16]. As MPOX is not endemic in Nepal and Nepali public is unfamiliar with it, most respondents scored poorly. Such poor knowledge is influenced by increasing age, low level of education, and the participants who did not use smart mobile phones for health-related information. These findings suggest that the massive awareness of public health programs on monkeypox is of utmost importance in Nepal among high-risk populations. The importance of awareness is already seen in the previous COVID-19 pandemic [17, 18].

MSM in the younger age group (<25 years) had a higher number of correct responses compared to those in older age groups. This finding is consistent with previous research conducted in Jordan, Nepal, and South Arabia, which also demonstrated a relationship between lower age and greater knowledge about COVID-19 [7, 13, 17]. It is possible that the increased use of social media and other online platforms among younger MSM contributed to their greater awareness of MPOX. In addition, persuasive communications among younger MSM may have facilitated the dissemination of information through peer networks.

It is challenging to pinpoint the exact reasons, but it appears that MSM who have completed high school education or its equivalent (i.e., plus two) possess greater knowledge of MPOX, which aligns with previous research conducted among the general population. [13, 19]. There might be many factors that could contribute to this relationship. For example, participants who pursued higher education might get more opportunities to learn about a variety of subjects, including topics related to MPOX. Additionally, MSM who invested the time and resources in their higher education might be more motivated to seek out and retain new knowledge. It is possible that the type of education or coursework that participants who completed at least high school education may have contributed to their knowledge of MPOX or infectious diseases, either directly or indirectly. MSM who studied plus two or above might get opportunities to participate in the awareness seminar, workshop, and training so they might have better knowledge of MPOX.

MPOX is believed to be a disease in African countries, but in 2022 it spread all around the globe, including Europe and America [4]. Individuals who use smartphones to access health information can easily find and learn about various health-related topics, including information about MPOX. By using smart mobile phones for health-related information, people can keep themselves informed about the latest developments and current events related to health topics, including MPOX [20]. Additionally, they might know that it is most common in MSM and might have studied more on it from their smartphone in a convenient way. Therefore, participants using smartphones for health-related information answered more correctly in comparison to those who did not, and this finding is supported by other studies. A previous study on the outbreak of MPOX found that working with affected communities and using digital media can help to provide updates on public health measures [7].

## Limitations and Recommendations

Several limitations must be considered when interpreting the results of this research. Firstly, since the study was cross-sectional, causation cannot be assigned to the results, and the findings may not be generalizable over time. In this regard, longitudinal research is crucial. Secondly, the study only included MSM participants from the Kathmandu valley, so the knowledge of general people and MSM living in other parts of Nepal could not be assessed. Thirdly, the study did not include questions about vaccine acceptance, which could have been helpful for stakeholders considering vaccination campaigns. Fourthly, the potential for response bias cannot be ruled out in this study, as some participants may have provided answers that were influenced by the researcher’s viewpoint. Finally, a robust infectious disease monitoring system is recommended to promote early illness identification and contact tracking.

## Conclusion

The outcomes of the present study revealed that the majority of MSM in Nepal had extremely poor knowledge regarding MPOX. However, according to the results, rapid health education initiatives and more accurate information should be given and disseminated to MSM by different health authorities. To lessen the risk of infection and spread of the virus, decision-makers and policymakers should take measures to improve MSM’s knowledge of MPOX. Since participants who used smartphones for health-related information responded more accurately, thus strengthened m-health, and intervention related to it among this population could be more fruitful.

## Figures and Tables

**Additional Figure:**
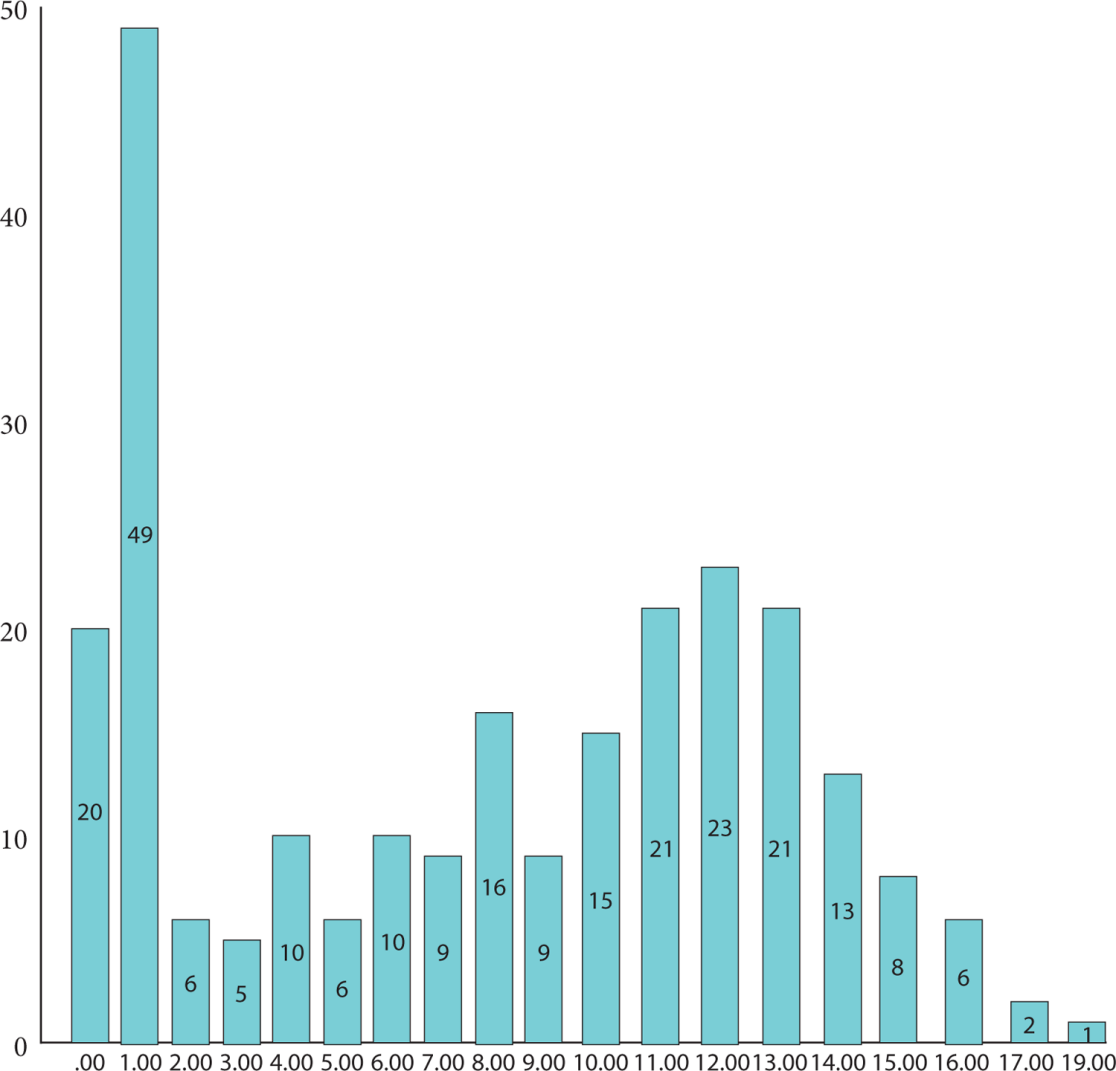
Total scores of human monkeypox knowledge. Respondents rated items as “Yes,” “No,” or “Don’t Know;” one score was awarded for a correct response and 0 for an incorrect answer or “Don’t Know.” The total score was 23. Human monkeypox knowledge items that are marked with blue color represent correct statements.

**Table 1:** General characteristics of the participants

Variables	Categories	Number (%)
**Age in years (Mean, SD)**		27.6 ±8.9
**Age**	Less than 25	127(50.8)
	25 and above	123(49.2)
**Sexual orientation**	Gay	158(63.2)
	Bisexual	92(36.8)
**Relationship status**	Single	161(64.4)
	With partner	89(35.6)
**Education**	Up to SLC	105(42)
	Plus two and above	145(58)
**Province of birth**	Bagmati	148(59.2)
	Outside Bagmati	102(40.8)
**Use of smartphones for health-relates information**	Never or rarely	91(36.4)
	Most of the time	159(63.6)

**Table 2:** The level of monkeypox knowledge among the study respondents divided age and education.

Monkeypox knowledge items	Response	Age (%)		P value	Education (%)		P value	Use of smartphones for health-related information		P value
		Less than 25	25 and above		Up to SLC	Plus two and above		Never or rarely	Most of the time	
What kind of disease does monkeypox cause ?	Correct	91 (51.7)	85 (48.3)	0.6	74 (42)	102 (58)	0.9	59 (33.9)	117 (66.5)	0.1
	Incorrect	36 (48.6)	38 (51.4)		31 (41.9)	43 (58.1)		32 (43.2)	42 (56.8)	
Monkeypox is a new infection that appeared this year 2022	Correct	27 (75)	9 (25)	0.002	9 (35)	27 (75)	0.02	7 (19.40)	29 (80.6)	0.02
	Incorrect	100 (46.7)	114 (53.3)		96 (44.9)	118 (55.1)		84 (39.3)	130 (60.7)	
Monkeypox is a sexually transmitted infection	Correct	29 (53.7)	25 (46.3)	0.6	15 (27.8)	39 (72.2)	0.01	11 (20.4)	43 (79.6)	0.006
	Incorrect	98 (50)	98 (50)		90 (45.9)	106 (54.1)		80 (40.8)	116 (59.2)	
Chickenpox and monkeypox are the same disease	Correct	67 (57.8)	49 (42.2)	0.04	31 (26.7)	85 (73.3)	<0.001	29 (25)	87 (75)	<0.001
	Incorrect	60 (44.8)	74 (55.2)		74 (55.2)	60 (44.8)		62 (46.3)	72 (53.7)	
Monkeypox is common in South Asian Countries?	Correct	33 (58.9)	23 (41.1)	0.2	20 (35.7)	36 (64.3)	0.3	13 (23.2)	43 (76.8)	0.02
	Incorrect	94 (48.5)	100 (51.5)		85 (43.8)	109 (56.2)		78 (40.2)	116 (59.8)	
Monkeypox is common in West and Central African countries	Correct	54 (49.1)	56 (50.9)	0.6	42 (38.2)	68 (61.8)	0.3	34 (30.9)	76 (69.1)	0.1
	Incorrect	73 (52.1)	67 (47.9)		63 (45)	77 (55)		57 (40.7)	83 (59.3)	
There are many cases recorded in Nepal.	Correct	36 (47.4)	40 (52.6)	0.5	30 (39.5)	46 (60.5)	0.6	19 (25)	57 (75)	0.01
	Incorrect	91 (52.3)	83 (47.7)		75 (43.1)	99 (56.9)		72 (41.4)	102 (58.6)	
Monkeypox cases are increasing in the USA and Europe	Correct	45 (48.4)	48 (51.6)	0.6	35 (37.6)	58 (62.4)	0.3	72 (41.4)	102 (58.6)	0.6
	Incorrect	82 (52.2)	75 (47.8)		70 (44.6)	87 (55.4)		32 (34.4)	61 (65.6)	
Monkeypox is a contagious viral disease.	Correct	64 (52.9)	57 (47.1)	0.5	44 (36.4)	77 (63.6)	0.08	59 (37.6)	98 (62.4)	0.3
	Incorrect	63 (48.8)	66 (51.2)		61 (47.3)	68 (52.7)		40 (33.1)	81 (66.9)	
Monkeypox is a contagious bacterial disease	Correct	15 (48.4)	16 (51.6)	0.8	10 (32.3)	21 (67.7)	0.2	51 (39.5)	78 (60.5)	0.03
	Incorrect	112 (51.1)	107 (48.9)		95 (43.3)	124 (56.6)		6 (19.4)	25 (80.6)	
Monkeypox is easily transmitted from one person to another.	Correct	15 (60)	10 (40)	0.3	13 (52)	12 (48)	0.3	85 (38.8)	134 (61.2)	0.7
	Incorrect	112 (49.8)	113 (50.2)		92 (40.9)	133 (59.1)		10 (40)	15 (60)	
Monkeypox is transmitted to humans through bites and scratches from an infected animal	Correct	47 (50.5)	46 (49.5)	0.9	39 (41.9)	54 (58.1)	0.9	81 (36)	144 (64)	0.6
	Incorrect	80 (51)	77 (49)		66 (42)	91 (58)		32 (34.4)	61 (65.6)	
People with monkeypox can transmit the disease to others (the disease is transmitted between humans).	Correct	64 (51.6)	60 (48.4)	0.8	49 (39.5)	75 (60.5)	0.4	59 (37.6)	98 (62.4)	0.8
	Incorrect	63 (50)	63 (50)		56 (44.4)	70 (55.6)		44 (35.5)	80 (64.5)	
Monkeypox is spread by droplets (coughing and sneezing).	Correct	35 (50)	35 (50)	0.8	35 (50)	35 (50)	0.1	47 (37.3)	79 (62.7)	0.7
	Incorrect	92 (51.1)	88 (48.9)		70 (38.9)	110 (61.1)		24 (34.3)	46 (65.7)	
The first symptoms of monkeypox are like the flu	Correct	54 (59.3)	37 (40.7)	0.04	32 (35.2)	59 (64.8)	0.09	67 (37.2)	113 (62.8)	0.8
	Incorrect	73 (45.9)	86 (54.1)		73 (45.9)	86 (54.1)		32 (35.2)	59 (64.8)	
Skin rash is a symptom of monkeypox	Correct	60 (48)	65 (52)	0.4	44 (35.2)	81 (64.8)	0.02	59 (37.1)	100 (62.9)	0.2
	Incorrect	67 (53.6)	58 (46.4)		61 (48.8)	64 (51.2)		41 (32.8)	84 (67.2)	
Monkeypox only affects males	Correct	58 (51.8)	54 (48.2)	0.8	38 (33.9)	74 (66.1)	0.02	50 (40)	75 (60)	0.1
	Incorrect	69 (50)	69 (50)		67 (48.6)	71 (51.4)		35 (31.3)	77 (68.8)	
Hand sanitizers and face masks are important in preventing monkeypox	Correct	48 (53.3)	42 (46.7)	0.5	39 (43.3)	51 (56.7)	0.7	56 (40.6)	82 (59.4)	0.9
	Incorrect	79 (49.4)	81 (50.6)		66 (41.3)	94 (58.8)		35 (31.3)	77 (68.8)	
There is a special treatment for monkeypox	Correct	18 (81.8)	4 (18.2)	0.002	6 (27.3)	16 (72.7)	0.1	56 (40.6)	82 (59.4)	0.6
	Incorrect	109 (47.8)	119 (52.2)		99 (43.4)	129 (56.6)		33 (36.7)	57 (63.3)	
Monkeypox is spread through bodily fluids	Correct	50 (53.2)	44 (46.8)	0.6	33 (35.1)	61 (64.9)	0.08	31 (33)	63 (67)	0.4
	Incorrect	77 (49.4)	79 (50.6)		72 (46.2)	84 (53.8)		60 (38.5)	96 (61.5)	
There is a monkeypox vaccine available in Nepal	Correct	26 (53.1)	23 (46.9)	0.7	20 (40.8)	29 (59.2)	0.09	17 (34.7)	32 (65.3)	0.8
	Incorrect	101 (50.2)	100 (49.8)		85 (42.3)	116 (57.7)		74 (36.8)	127 (63.2)	
The chickenpox vaccine I got in childhood protects me from monkeypox	Correct	36 (55.4)	29 (44.6)	0.3	20 (30.8)	45 (69.2)	0.02	15 (23.1)	50 (76.9)	0.009
	Incorrect	91 (49.2)	94 (50.8)		85 (45.9)	100 (54.1)		76 (41.1)	109 (58.9)	
There is a smallpox vaccine that can be used for monkeypox	Correct	17 (56.7)	13 (43.3)	0.4	15 (50)	15 (50)	0.02	10 (33.3)	20 (66.7)	0.7
	Incorrect	110 (50)	110 (50)		90 (40.9)	130 (59.1)		81 (36.9)	139 (63.2)	

## Data Availability

The data supporting the article will be made available by the authors upon reasonable request.
